# Numerical simulation of temperature field of a new type of freezing device under seepage effect

**DOI:** 10.1371/journal.pone.0298003

**Published:** 2024-05-16

**Authors:** Ning Yang, Xuyong Wang, Jixun Ren

**Affiliations:** 1 School of Architecture and Construction, Jiangsu Vocational Institute of Architectural Technology, Xuzhou, China; 2 JiangSu Collaborative Innovation Center for Building Energy Saving and Construct Technology, Xuzhou, Jiangsu, China; 3 Qingdao Geologic and Mineral Geolechnical Engineering Co.,Ltd., Qingdao Shandong, China; 4 School of Civil Engineering and Architecture, Hainan University, Haikou, China; Tribhuvan University, NEPAL

## Abstract

In order to investigate the development of the temperature field of a new type of freezing reinforcement under seepage conditions, in this paper, COMSOL finite element software was used to simplify the model and simulate the effect of groundwater seepage on the development of the temperature field of frozen pipes by coupling the Darcy’s law module and the heat transfer module for porous media. The heads of water were also varied to simulate the change in seepage velocity to further investigate the effect of seepage velocity on the temperature field. The results of the study show that the freezing wall formed in the high head region was thinner than that in the low head region due to the effect of seepage, and this phenomenon was aggravated with the increase of seepage rate; The effect of seepage action on the temperature field had a hysteresis along the seepage direction; When the seepage rate was greater than 1.65 m/d, the soil in the center of the device feezed better and could form a tight and dense freezing wall comparable to the size of the freezing device; When the seepage rate was greater than 5.78 m/d, the temperature of the center soil body gradually increased, and eventually the freezing curtain cannot be formed.

## 1. Introduction

Artificial freezing technology was proposed by a German engineer in the 1860s and was initially applied in mine excavation for mining coal [1, 2]. It has been widely used abroad due to its advantages of safety, flexibility and efficiency [3]. Since the 20th century, many countries have used artificial ground freezing technology in engineering fields such as subways, tunnels and coal mines, and have accumulated a lot of experience [4–8]. Artificial ground freezing technology was first introduced in China in the 1950s, and after more than 40 years of development, the technology is now widely used in various engineering fields such as foundation reinforcement and subway tunnels in China [9, 10]. Most previous studies on the development of artificial freezing temperature fields based on ADINA finite element software, which didn’t take into account the influence of groundwater seepage over temperature field [11–14]; In recent years, the simulation study of the development of freezing temperature field by using the hydrothermal coupling module of COMSOL software under the consideration of seepage is widely used in China and abroad. Takashi T [15] was the first to study the influence of groundwater seepage over temperature field, using Duthie’s law and other thermodynamic equation to explain it, but did not derive a formula; Vitel M et al. [16] have carried on the numerical simulation research to the temperature field and the seepage field, which had carried on the verification through the experiment. Domestic scholars such as Pan Xudong et al. [17] studied the influence of seepage field on the temperature in the construction of water-rich strata freezing method with relevant engineering application examples; Xiao Wei et al. [18] studied the mutual influence of seepage field and temperature field in different groundwater flow system modes; Ye Chao et al. [19] studied the influence of groundwater salinity on freezing temperature field. Most of the above domestic scholars only established two-dimensional numerical models for discussion, the study was not comprehensive.

Based on the above status, this paper combines previous research results to conduct a numerical simulation study on a new subsea freezing device [20] with hydrothermal coupling. Simplified the complex device and soil into a simple heat transfer model, considered both temperature and seepage conditions. Established a three-dimensional visualization numerical model, and discussed the evolution law of temperature field under different heads of water (seepage velocity). Sets up three paths for comparative analysis, explores the freezing effect of the device, and provides relevant references for the application of the device in soil reinforcement in the marine environment.

## 2. Simplification of the research object

**Fig 1:**
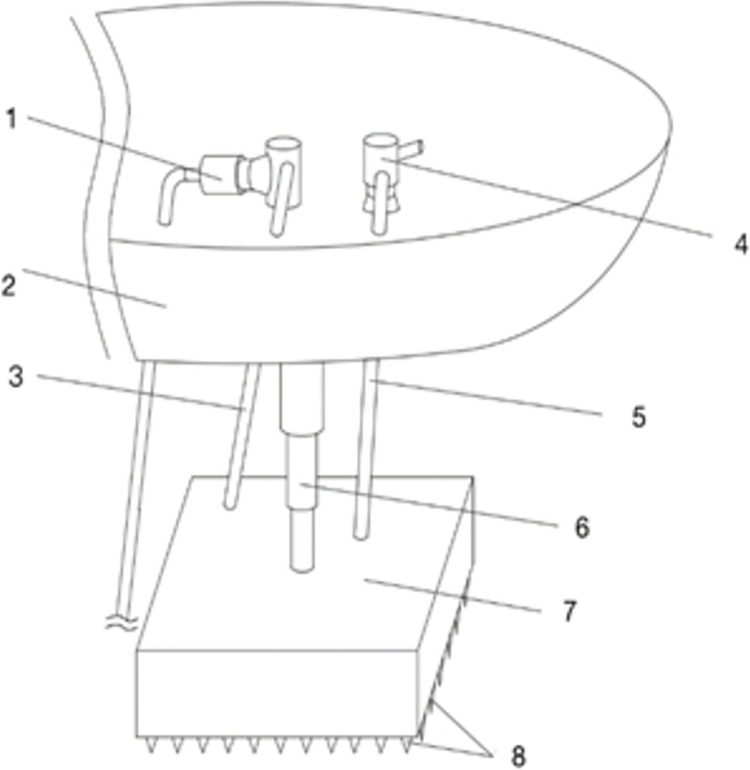
Schematic diagram of the exterior of the freezing unit. 1. Sand pump; 2. Operation boat; 3. Sand pumping pipe; 4. Pumping pump; 5. Pumping pipe; 6. Hydraulic telescopic rod; 7. Tank box; 8. Bump.

**Fig 2:**
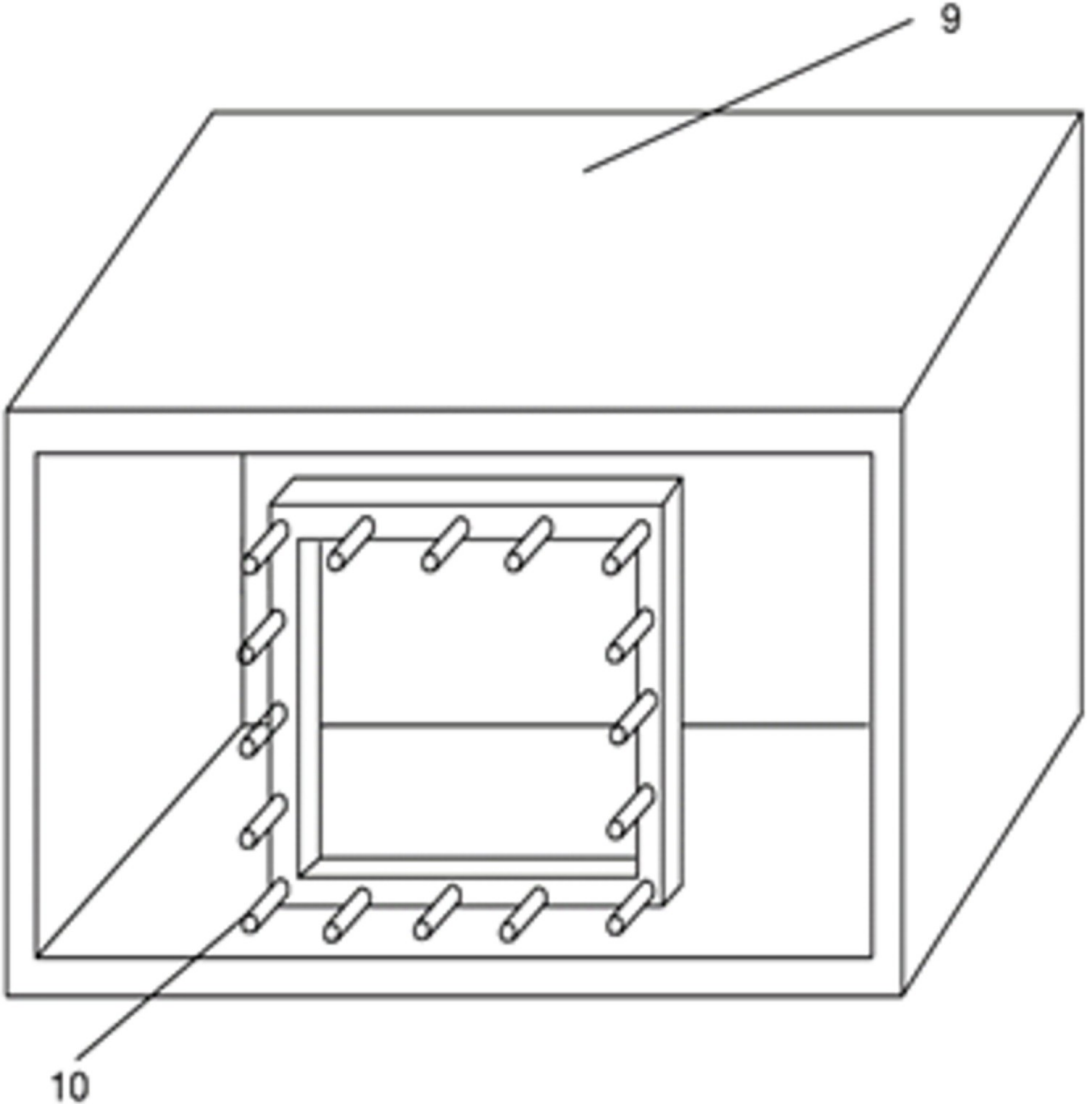
Schematic diagram of the interior of the artificial freezing unit. 9. Tank box; 10. Freeze pipe.

**Fig 3:**
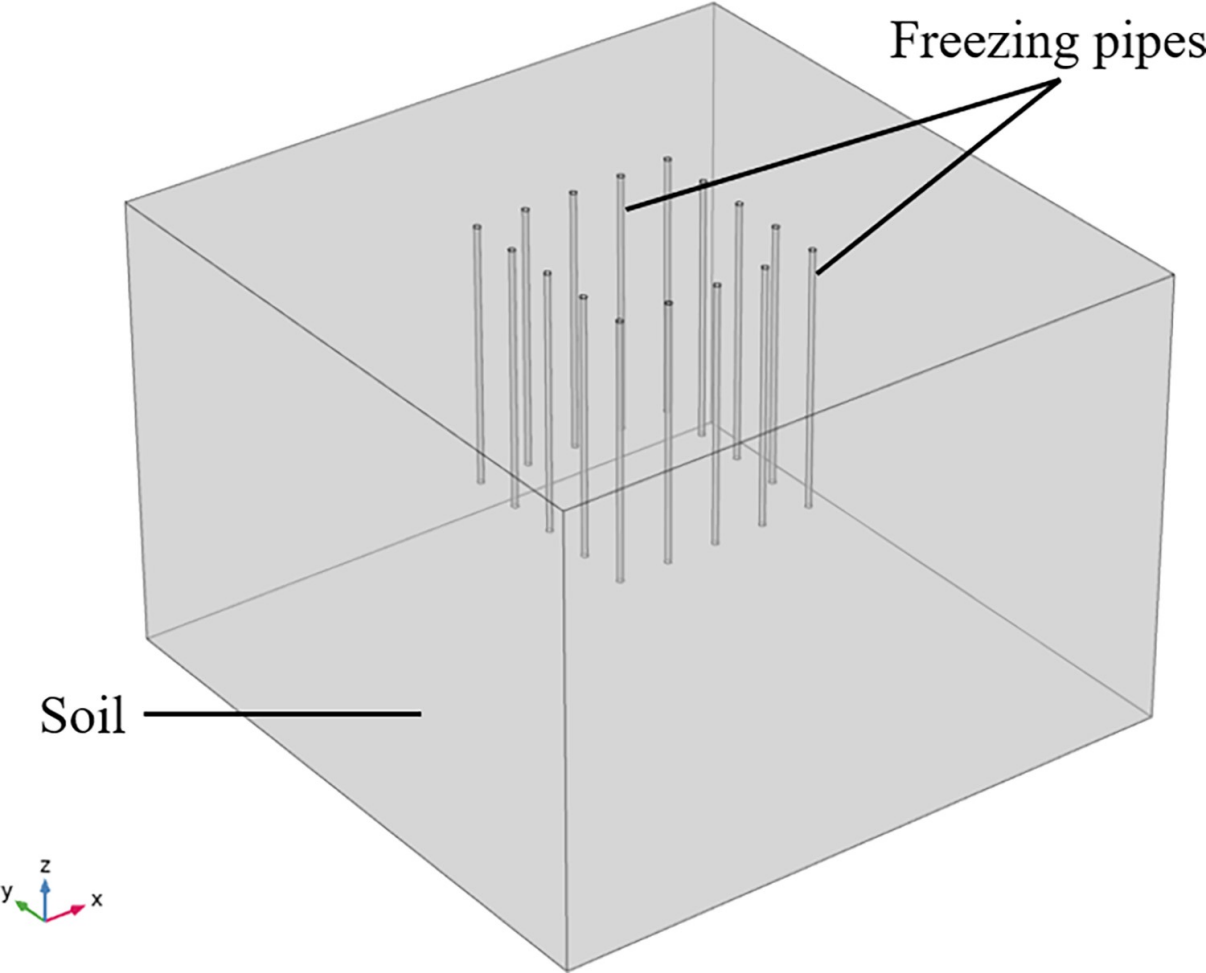
Simplified model.

This underwater artificial freezing device, as shown in Figs 1 and 2, includes an offshore operating vessel, a hydraulic telescopic rod, a sealed tank box, a freezing mechanism, and a water pumping mechanism, and the freezing mechanism includes a rectangular frame, a connecting rod, a freezing tube body, a brine pump, a refrigeration sheet, and a brine tank. The pumping system includes a pump and a pumping pipe. After moving to the area to be constructed by the marine operation vessel, the sealing tank box is driven by the hydraulic telescopic rod to descend to the area to be constructed of the submarine soil, and the sealing tank box forms a sealed space above the submarine soil, and the seawater in the sealed space is pumped out by the pump, and then the low-temperature brine is delivered to the freezing pipes by the brine pump so that the freezing pipes can freeze the submarine soil and form a permafrost curtain. In this paper, we only consider the effect of freezing pipes heat transfer on the soil, so the complex structure of the upper part of the freezing device was omitted and simplified to a simple freezing pipes heat transfer model as shown in Fig 3 with the following assumptions:

(1) Ignoring the inhomogeneous distribution of the submarine soil, the submarine soil is assumed to be a regular rectangular body in the numerical simulation;

(2) Assume that the frozen pipes construction process was vertical and did not produce frost heave deflection and did not produce displacement during the freezing process;

(3) Neglecting the force of the submarine soil on the frozen pipes, the frozen pipes are assumed to be rigid and the load was uniformly distributed on the surface of the frozen pipes.

## 3. Theoretical analyses

This study is closely related to the temperature and seepage fields, so it is necessary to use COMSOL software to simulate the coupling of temperature and seepage fields, and the temperature changes transiently during the simulation, without considering the influence of stress and strain on the simulation results during the freezing process, and the freezing of sandy soil under seepage conditions is regarded as a heat transfer seepage model.

### 3.1 Seepage field coupling

During freezing, seawater seepage conforms to Darcy’s law. Its equation is expressed as [21]:

$$Q=K∙A∙\frac{\Delta H}{L}$$ (1)

where *Q* denotes the seepage volume; *K* denotes the hydraulic conductivity of porous media in m/s. *A* denotes the cross-sectional area perpendicular to the seepage direction, Δ*H* denotes the head of water, L denotes the seepage path.

In COMSOL software, Darcy’s law is used to analyze groundwater seepage with its own partial differential equation as [21]:

$$\frac{\partial }{\partial_{t}}(\epsilon_{p}\rho )+\nabla ∙(\rho u)=Q_{m}$$ (2)

$$v=\frac{K∙\Delta H}{L}$$ (3)

Where *ϵ*_*p*_ denotes porosity rate; *Q*_m_ denotes various source terms; *v* denotes the speed field in m/s; *K* denotes the hydraulic conductivity of porous media in m/s.

In Eq (3), the permeability coefficient K changes with temperature during freezing, and the permeability coefficient is a function of temperature. Therefore, the coupling of the temperature and permeability fields can be expressed as a functional expression. The Heaviside step function is used for the water-ice phase change during freezing [21]:

$$K(T)=K_{U}H(T)+K_{f}(1-H(T))$$ (4)

where *K*_*f*_ and *K*_*u*_ denote the permeability coefficients of unfrozen sand and frozen sand, respectively, in m/s; in *H(T) = flc2hs(T-T*_*d*_,*δT)*, T denotes the temperature of the sandy soil, *T*_*d*_ denotes the temperature of the phase change point, and *δT* denotes the radius of the phase change zone.

### 3.2 Temperature field coupling

It is assumed that the sand is saturated, homogeneous, and isotropic during the freezing process, and the temperature field is affected by the heat transfer from the porous medium and the seepage heat transfer from the seawater. In the freezing process, the volume ratio of seawater and porous media particles can be expressed as *(1 - θ*_*S*_*)/θ*_*S*_, and the differential equation of the temperature field in the heat transfer process is [21]:

$$C_{eq}\frac{\alpha T}{\alpha \tau }=\lambda_{eq}(\frac{\alpha^{2}T}{\alpha x^{2}}+\frac{\alpha^{2}T}{\alpha y^{2}}+\frac{\alpha^{2}T}{\alpha z^{2}})-(\rho_{w}C_{w})(V_{x}\frac{\alpha T}{\alpha x}+V_{y}\frac{\alpha T}{\alpha y}+V_{z}\frac{\alpha T}{\alpha z})$$ (5)

Where T denotes temperature; τ denotes time; *C*_*eq*_ denotes the equivalent volumetric heat capacity of saturated sand; *λ*_*eq*_ denotes the equivalent thermal conductivity; *ρ*_*w*_ denotes the density of water; *C*_*w*_ denotes the specific heat of water; *V*_*x*_, *V*_*y*_ and *V*_*z*_ denote the percolation velocity of water.

The pore medium heat transfer module in COMSOL software is used to analyze the heat transfer problem, and its own equation is [21]:

$${\left(\rho C_{p}\right)}_{eff}\frac{\alpha T}{\alpha t}+\rho C_{p}u∙\nabla T+\nabla ∙q=Q+Q_{vd}$$ (6)

$$q=-k_{eff}\nabla T$$ (7)

$${\left(\rho C_{p}\right)}_{eff}=C_{eq}$$ (8)

$$k_{eff}=\lambda_{eq}$$ (9)

where *∇* denotes the Nabla operator; *k*_*eff*_ denotes the equivalent thermal conductivity; and *Q* and *Q*_*vd*_ denote various source terms.

When the saturated sand freezes the water freezes to produce latent heat of phase change, which will change the physical parameters of frozen sand, and the temperature change during freezing can be regarded as a transient heat transfer process.If the temperature of the soil particles around the pore is lower than the temperature of the liquid in the pore, according to the conclusion of heat transfer, due to the temperature difference, heat transfer occurs between the liquid and the solid, and the liquid outputs heat to the surrounding solid through convective heat exchange, forming an ice phase change in the pore water to complete the freezing process. Therefore, the heat balance equation considering the phase change case is established as [21]:

$$C_{eq}\frac{\alpha T}{\alpha \tau }+\rho_{w}L\frac{\alpha \theta_{W}}{\alpha T}+\nabla (\rho_{w}C_{w}uT-\lambda_{eq}\nabla T)=Q$$ (10)

where *L* denotes the latent heat of phase change of water freezing; *θ*_*w*_ denotes the content of flowing water per unit volume; and *Q* denotes the heat source.

### 3.3 The coupling of the seepage and temperature fields

There are two main aspects. On the one hand, the percolation field in the porous media system changes, which can lead to the permeate water flow participating in the heat transfer and exchange in the porous media system, making the temperature field affected. On the other hand, the temperature field in the spongy media system changes, and the temperature difference generated will cause the water to change leads to the change of the permeability coefficient of the spongy media system. However, the modulus of elasticity and strength of the soil are functions of the temperature, so the change of temperature will also lead to the change of the permeability field of the porous media system.

## 4. Construction of the numerical model

### 4.1. Numerical geometry modeling and path setup

**Fig 4:**
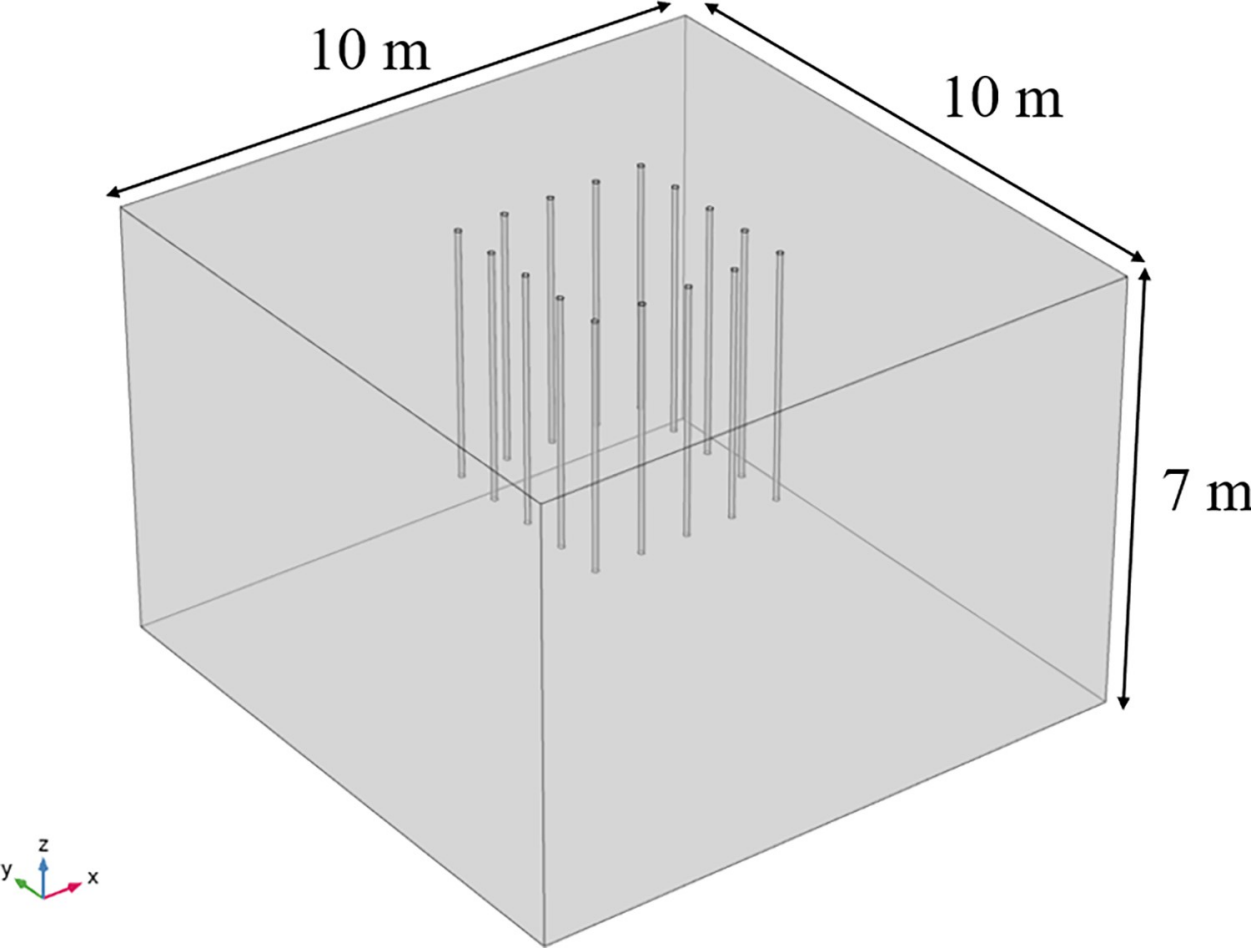
3D soil model.

**Fig 5:**
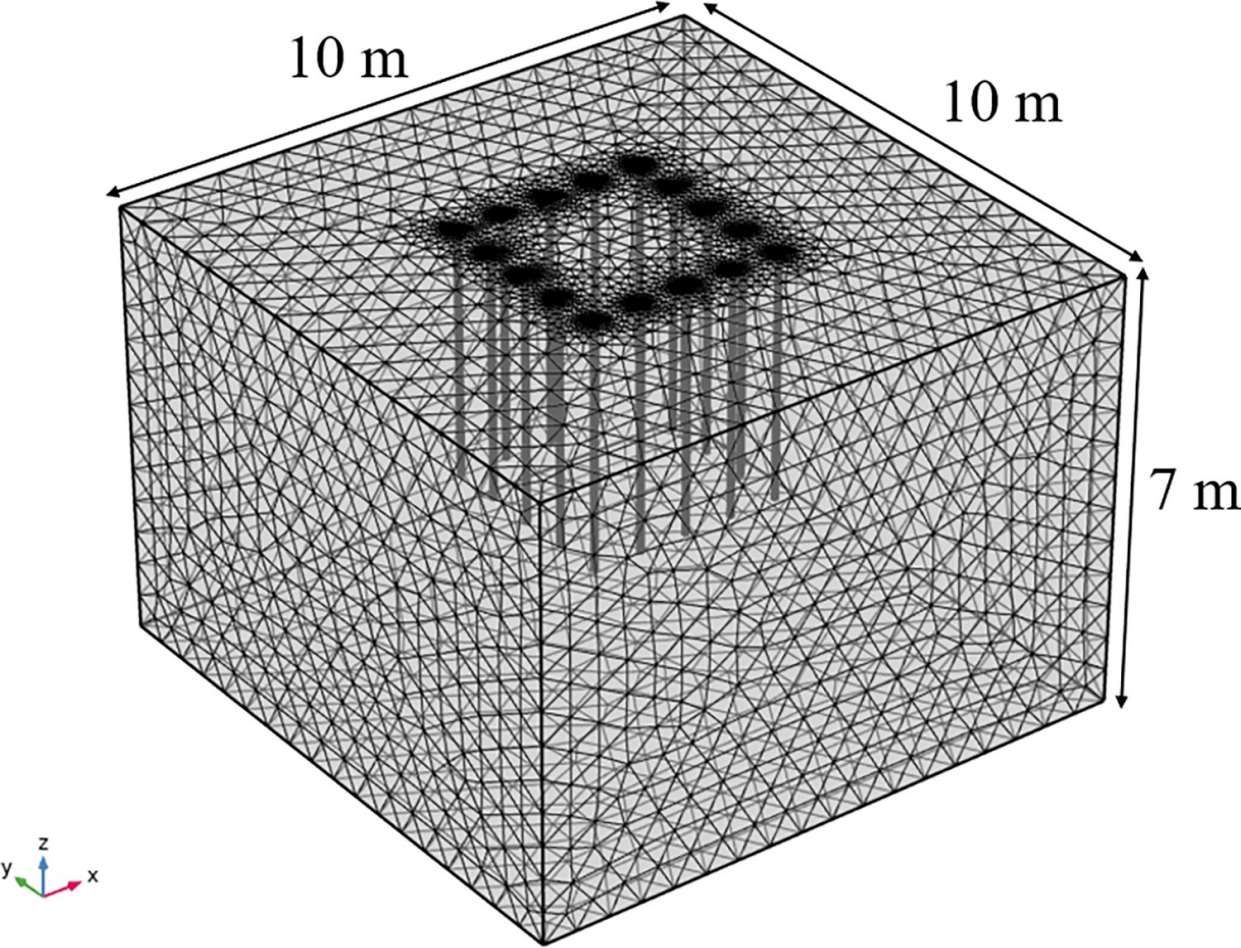
Schematic diagram of meshing.

Transient 3D heat transfer model with phase change using COMSOL Multiphysics 5.4, and the geometric model dimensions are determined empirically. The geometric dimensions are: take the X-axis direction as the length, Y-axis direction as the width, and Z-axis direction as the depth, which are 10 m×10 m×7 m respectively. Through numerical simulation and calculation analysis, the freezing influence range is stable and reliable for the calculated structure in this area. The freezing pipe’s radius is 0.05 m, the spacing is 0.8 m, and the length is 4 m. A total of 16 pipes are arranged in a square arrangement, and the geometric model is shown in Fig 4. Triangular mesh division is used to improve convergence and refine the model to accurately study the effect of seepage on the temperature field, and the mesh division is shown in Fig 5. According to the research of related scholars, it is feasible to use COMSOL hydrothermal coupling to numerically simulate the development law of temperature field under seepage conditions. Similar studies such as Hu Zhuang [22] and others on the study of the freezing method construction temperature field evolution law of the contact channel of the Nanning Metro Xin Guang interval, comparing the model calculation results with the engineering actual measurement data, can reflect the actual engineering profile more realistically.

**Fig 6:**
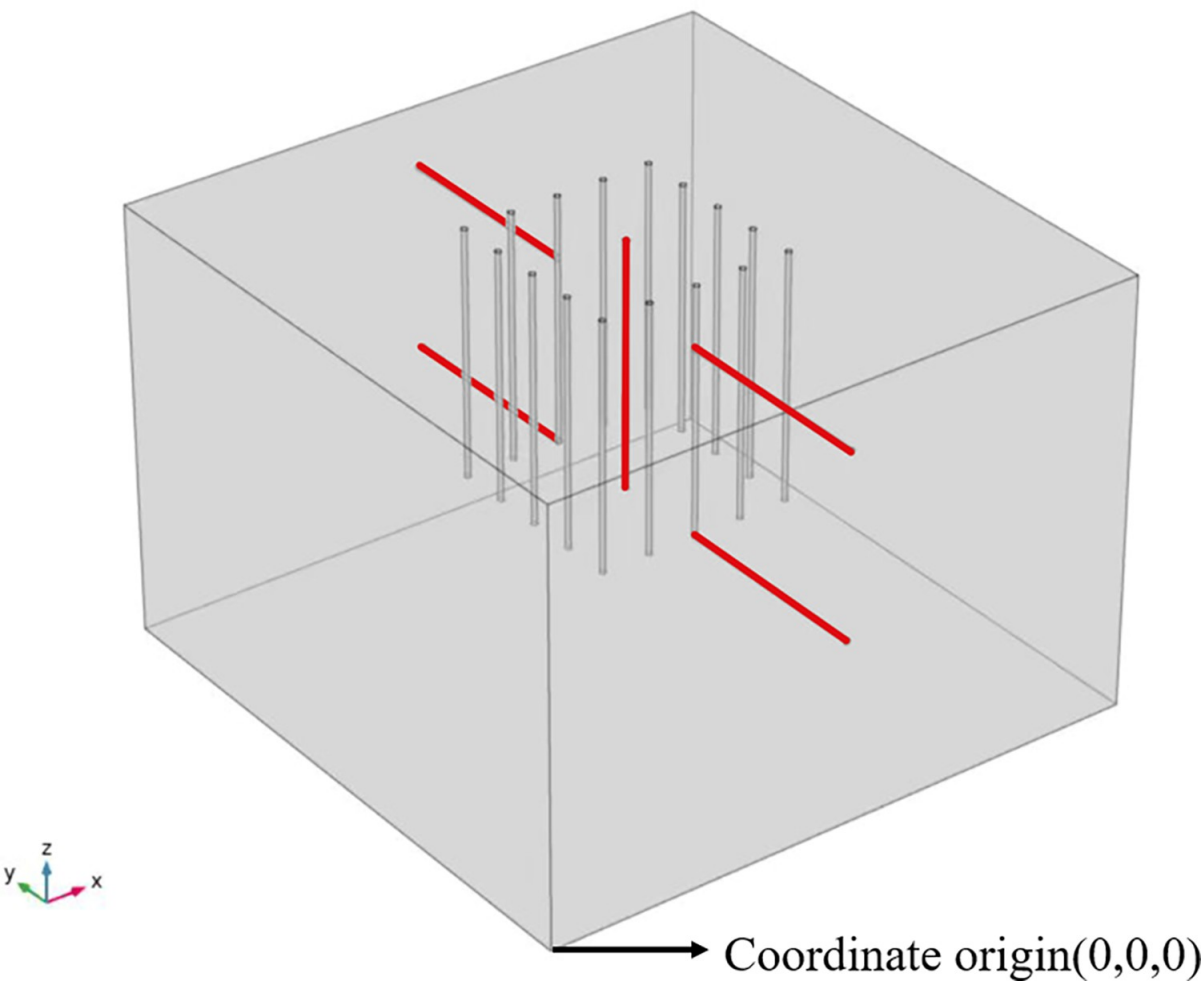
3D path diagram.

**Fig 7:**
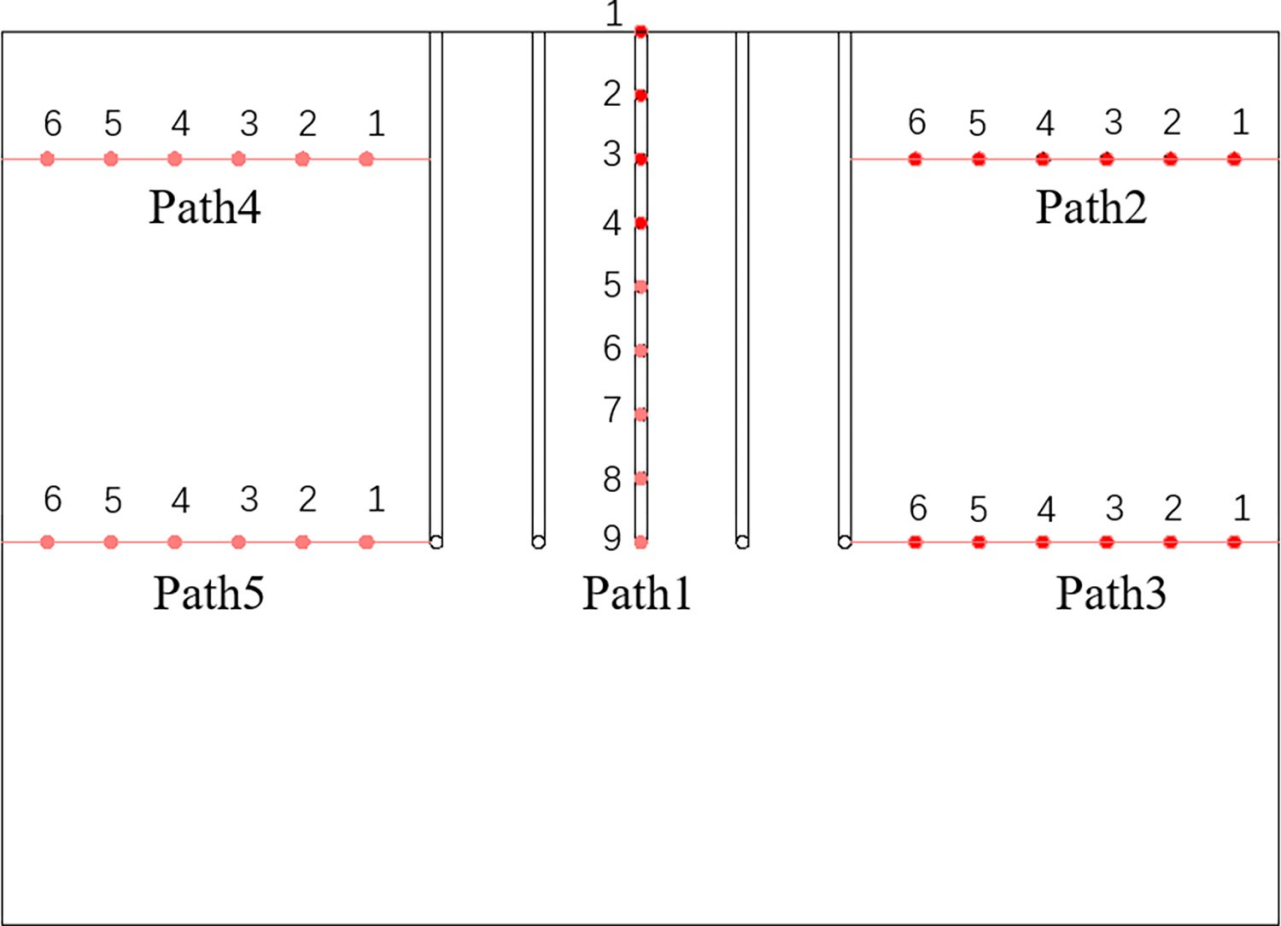
YZ (X = 0) cross-sectional path diagram.

In this paper, five observation paths are set up, as shown in Figs 6 and 7. The coordinate origin of the numerical model was (0, 0, 0), as shown in Fig 6. To explore the freezing of the geometric center of the soil, a path 1 perpendicular to the XY plane with starting and ending coordinates (5, 5, 7) and (5, 5, 3) were set up. One observation point was set up every 0.5 m, and a total of 9 were set up; To investigate the variation pattern of the temperature field in the seepage direction, a path2 was set perpendicular to the flow plane, which was perpendicular to the XZ plane with the starting and ending coordinates of (5, 3.4, 6) and (5, 0.4, 6); an observation point was set every 0.5 m, and a total of 6 were set. To investigate the difference in the temperature field along the depth (Z-axis) in the seepage direction, a path 3 parallel to path 2 was set up with starting and ending coordinates (5, 3.4, 3) and (5, 0.4, 3). An observation point was set up every 0.5 m, and a total of 6 were set up. To investigate the effect of seepage in the upstream region on the temperature field, paths 4 and 5 were set up symmetrically with paths 2 and 3, starting and ending at coordinates (5, 6.6, 6), (5, 9.6, 6), (5, 6.6, 3), and (5, 9.6, 3), respectively. One observation point was set up every 0.5 m, and six were set up for each path. In this paper, a total of 33 observation points were set up.

### 4.2. Initial and boundary conditions

#### 4.2.1. Initial conditions

The initial temperature of the submarine sandy soil is assumed to be 291.15 K (18°C), and the initial temperature of the porous medium is also 291.15 K (18°C) for the numerical calculation. The relationship between seepage velocity and seawater flow rate in the submarine sandy soil is [21]:

v=nV (11)

Where *v* denotes the seepage velocity in m/s; *n* denotes the porosity; *V* denotes V is the microscopic fluid velocity in the pore space.

The relationship between head difference and seepage velocity can be expressed as [21]:

$$K=\frac{k\rho g}{\eta }$$ (12)

Where *K* denotes the hydraulic conductivity of porous media in m/s; k denotes the permeability of porous media in m^2^; *ρg* denotes the gravity in N/m^3^; *η* denotes the dynamic viscosity in N·s/m^2^.

#### 4.2.2 Boundary conditions

**Fig 8:**
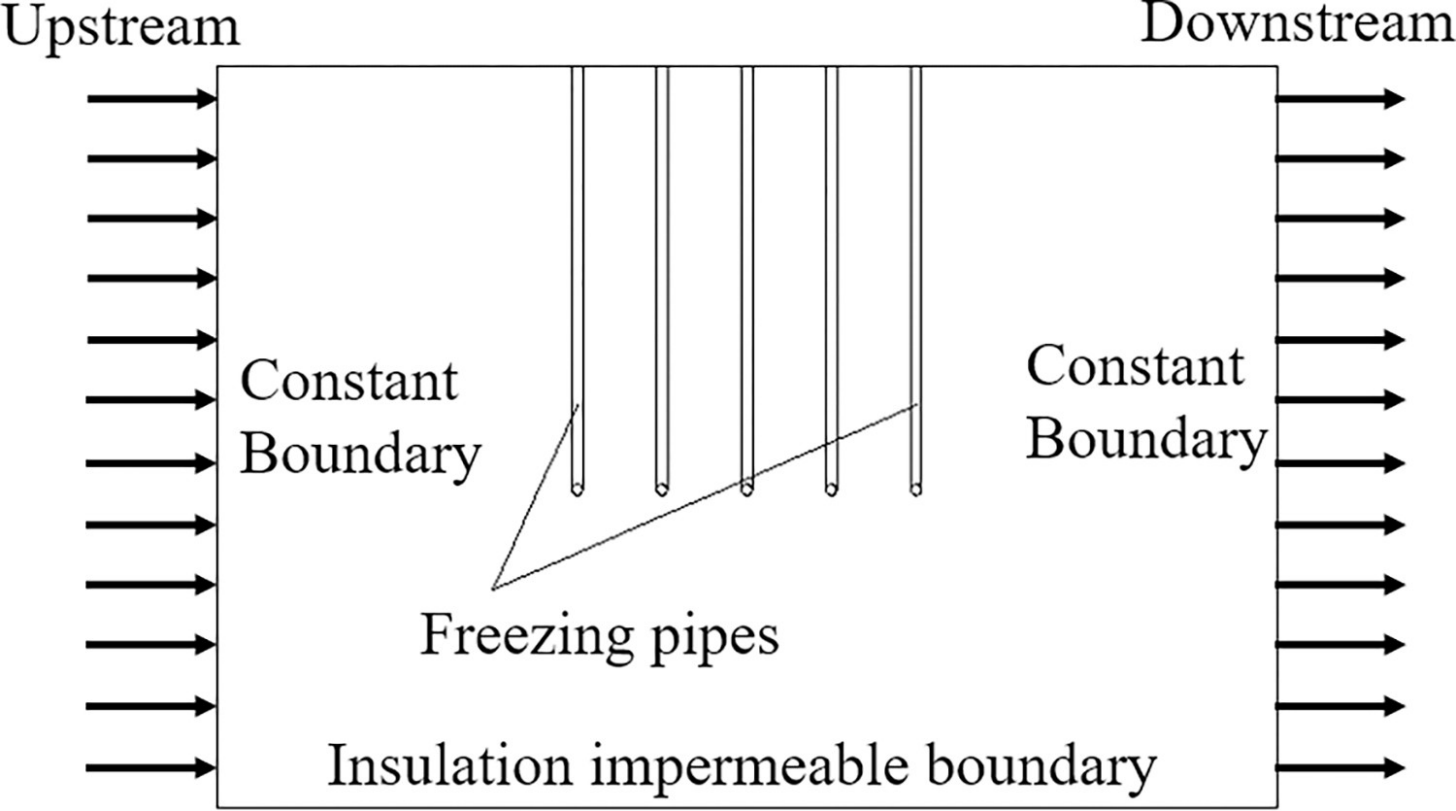
Boundary conditions (YZ section).

All six faces of the numerical model are set as adiabatic, and the XZ(Y = 10) plane is taken upstream and the XZ(Y = 0) plane is taken downstream, two planes are set as constant head, and the other four planes and the edge of the frozen pipe are set without flow, and the boundary conditions are shown in Fig 8.

### 4.3. Parameters selection

The study of the coupling of seepage and temperature fields involves parameters of water, ice, sand and frozen sand, such as density, thermal conductivity, specific heat, permeability coefficient, porosity, etc., and each physical parameter was obtained from permafrost tests [23, 24] as shown in Table 1.

**Table 1:** Physical parameters.

Name of the Parameters		Value
The density of water (kg/m^3^)		1000
The density of ice (kg/m^3^)		917
The density of soil (kg/m^3^)	Unfrozen soil	1920
	Frozen soil	1895
Thermal conductivity of water (W/(m∙K))		0.63
Thermal conductivity of ice (W/(m∙K))		2.31
Thermal conductivity of soil (W/(m∙K))	Unfrozen soil	1.72
	Frozen soil	1.96
Specific heat of water (J/(kg∙K))		4180
Specific heat of ice (J/(kg∙K))		2050
Specific heat of the soil (J/(kg∙K))	Unfrozen soil	1690
	Frozen soil	1690
Hydraulic conductivity of the soil (m/s)	Unfrozen soil	1.91×10^−4^
	Frozen soil	1.16×10^−30^

Some physical parameters of the sandy soil change with temperature during active freezing of the model. Among them, the density, thermal conductivity, specific heat, and permeability coefficient change most significantly. Therefore, only the thermal conductivity, specific heat capacity, and permeability coefficient are considered in the numerical modeling with temperature. In COMSOL definition of material parameters select segmentation function, 272.15 K to 303.15 K (-1°C to 30°C) for the unfrozen temperature, 243.15 K to 272.15 K (-30°C to -1°C) for the frozen temperature, the two temperature intervals physical parameters are not the same, as shown in Table 1. The permeability coefficient of unfrozen sandy soil was determined by physical experiments, and when the soil temperature reaches 272.15 K (-1°C) or less, the visibly frozen sandy soil is completely impermeable, so the permeability coefficient of frozen sandy soil can be taken as 1.16×10^−30^ m/s. The permeability k of the porous medium needs to be entered when defining the material in COMSOL. Therefore, K needs to be converted to k with the help of Eq 12.

In the numerical simulation process, the interpolation function h(t) was used to represent the freezing pipes cooling plan, and the number of freezing days was set to 40 d according to the construction experience, and the calculation step was 24 h. The freezing pipes cooling plan is shown in Table 2 [21].

**Table 2:** The freezing pipes cooling plan.

Time (Day)	0	1	5	10	20	30	40
**Temperature (°C)**	18	0	−20	−25	−28	−28	−28

### 4.4. Basic assumptions

Assuming that the sandy soil is a homogeneous continuous porous media soil, and the initial temperature of both the sandy soil and the groundwater temperature are consistent at 18°C;

Assume that the water infiltration process is unidirectional and uniform, that there is no energy loss in the freezing process, and that no energy is transferred outward from the frozen area;

Assume that the brine temperature is the same as the temperature of the outer wall of the frozen pipe, ignoring the heat loss caused by convective heat transfer of brine;

Assuming that the seepage field within the frozen area conforms to Darcy’s law and that the seepage velocity is close to zero after the soil freezes;

Assuming that the stress field has no effect on the temperature field and that the freezing process considers only the coupling effect of the temperature and seepage fields;

Assume that the temperature will be -1°C when the freezing wall starts to form, and the freezing wall at -10°C meets the construction requirements.

## 5. Calculation results and analysis

**Fig 9:**
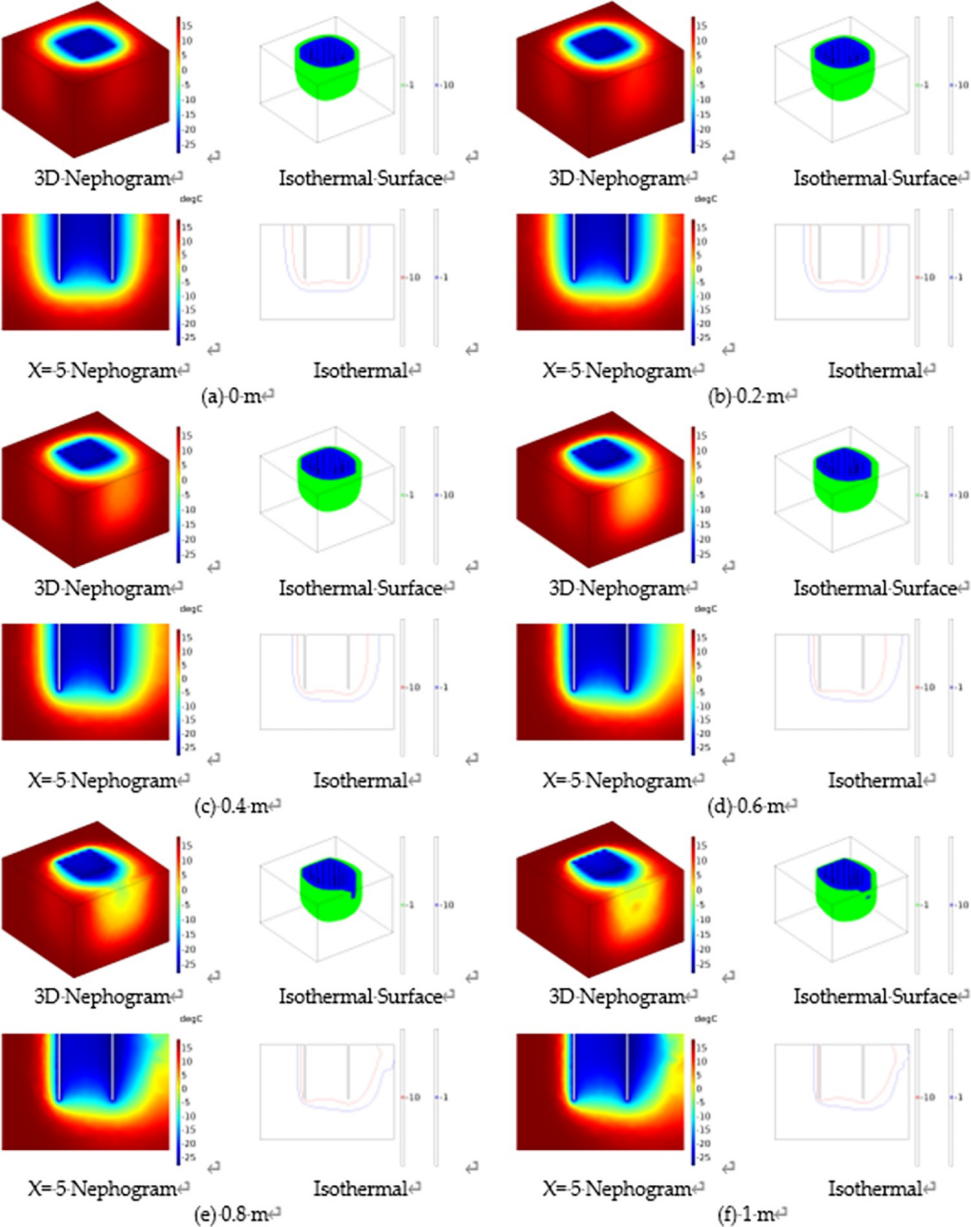
Temperature development diagram for different heads of water.

To investigate the development of the temperature field of the new freezing device at different seepage velocities, based on the actual survey data from the National Earth System Science Data Center, the heads of water between upstream and downstream of a sea in the South China Sea is between 0.2 and 1 m. On this basis, the effect of seepage velocity on the temperature field of the new freezing device is simulated by establishing numerical models with different head differences, and the heads of water and seepage velocity are calculated to be positively related according to Eq (12), specifically The numerical values are shown in Table 3. Under the condition that other physical fields and external conditions are consistent, the temperature field development under different seepage velocities is simulated by changing the heads of water between the upstream and downstream of the model to explore the effect of seepage velocity on the temperature field, and the results are shown in Fig 9.

**Table 3:** Brine cooling plan.

Heads of water (m)	0	0.2	0.4	0.6	0.8	1.0
**Seepage velocity (m/d)**	0	0.33	0.66	0.99	1.32	1.65

**Fig 10:**
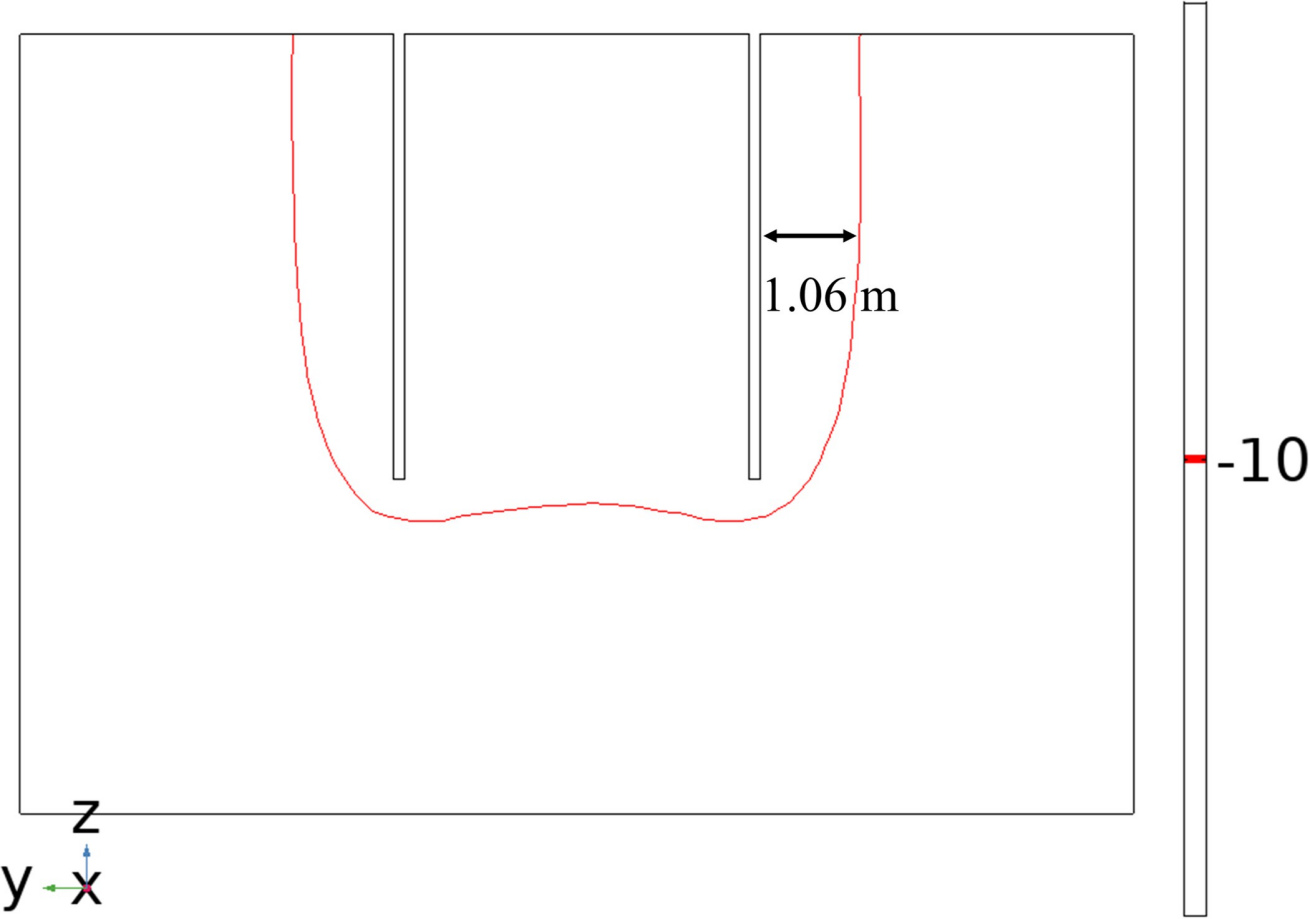
Thickness of permafrost curtain.

**Fig 11:**
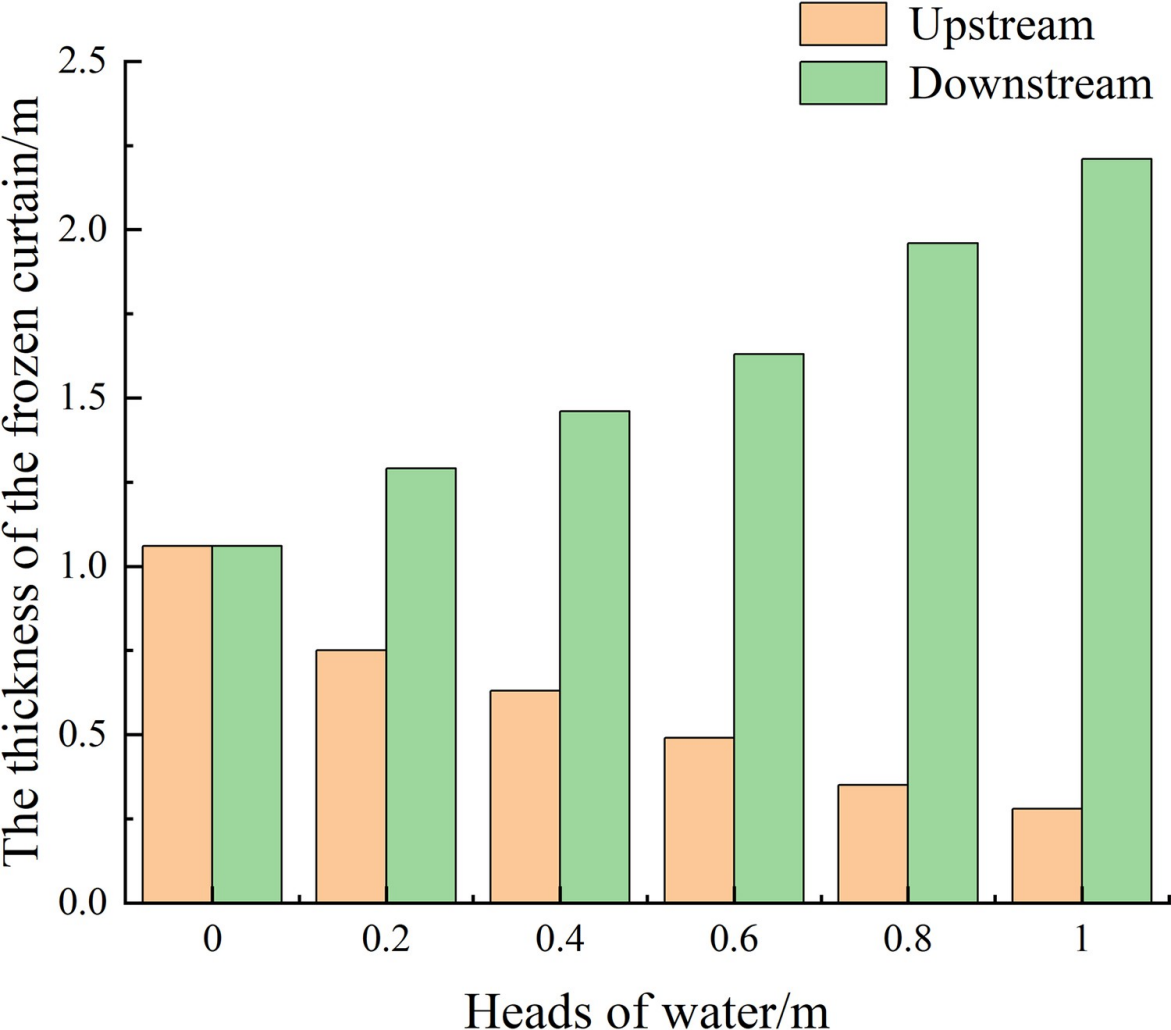
Variation of permafrost curtain thickness with heads of water.

After 40 days of active freezing, it can be seen from Fig 9 that the permafrost curtain was symmetrically distributed on both sides of the frozen pipes with uniform thickness when the seepage velocity was 0. By measuring the distance between the 236.15 K (-10°C) isotherm and the edge of the frozen pipe, it was found that the thickness of the tight and dense frozen wall was about 1.06 m, as shown in Fig 10. The temperature in the center of the soil body is lower than the surrounding temperature; when seepage flow exists, the thickness of the frozen curtain in the upstream region becomes significantly thinner and thinner with the increase of seepage velocity, and the asymmetry of the frozen curtain increases and gradually shifts to the downstream region. The relationship between seepage velocity and permafrost curtain thickness is shown in Fig 11.

**Fig 12:**
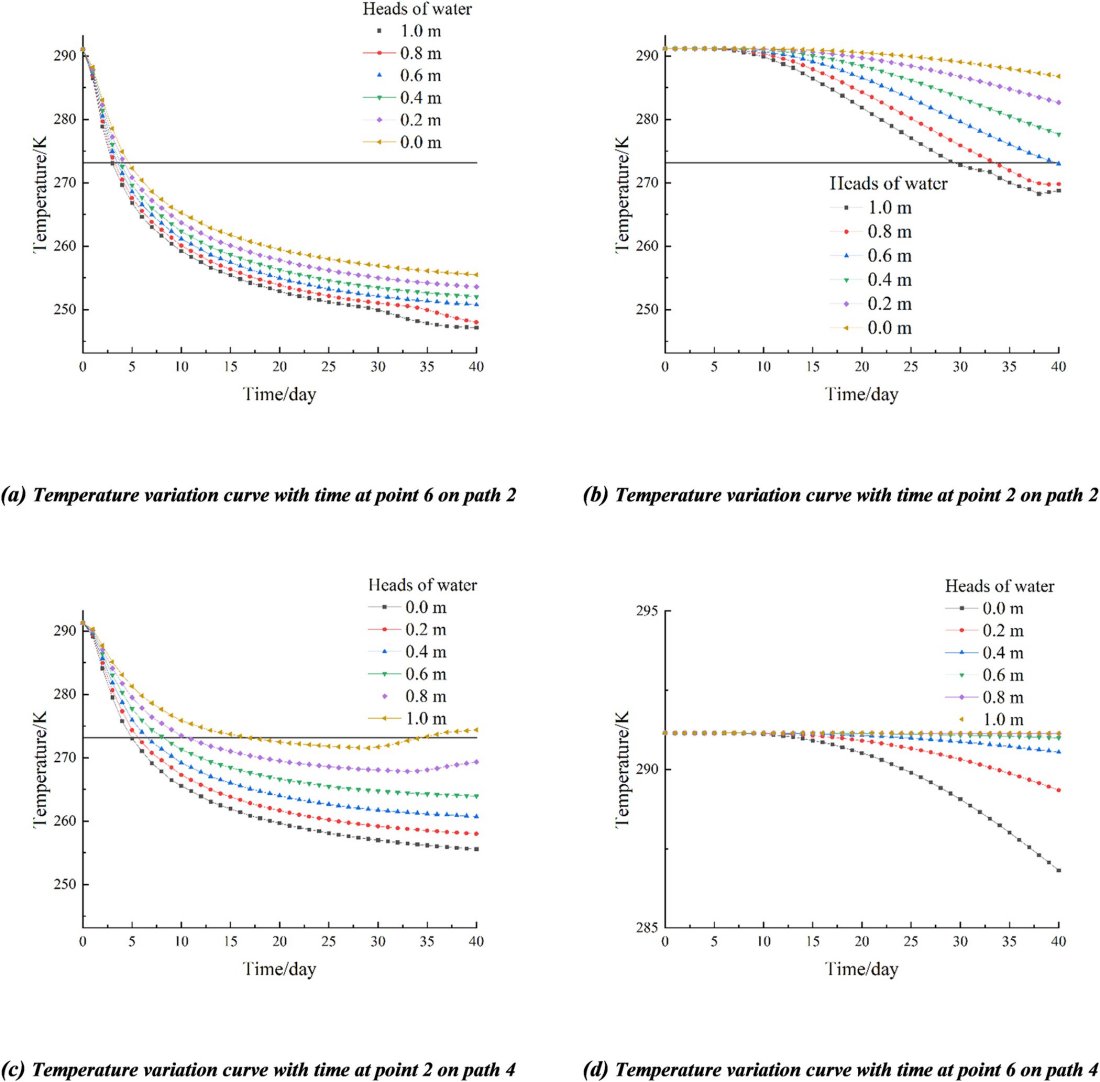
Temperature variation curve of observation point with time.

XZ (Y = 10) upstream and XZ (Y = 0) downstream. A total of 4 observation points were taken at symmetrical positions upstream and downstream, 2 of which were closer to the freezing pipes and 2 of which were farther away. Therefore, observation points 2 (5, 2.9, 6) and 6 (5, 0.4, 6) on path 2 and observation points 2 (5, 7.1, 6) and 6 (5, 9.4, 6) on path 4 were selected, and the temperature variation curves of the observation points with time are shown in Fig 8. As can be seen in Fig 12(A), the soil closer to the frozen pipes cools rapidly from the first day and reaches a temperature of 272.15 K (-1°C) on approximately day 5 for a percolation rate of 0 in the downstream region. With the increase of seepage velocity the cooling rate of soil is accelerated. From Fig 12(B), it can be seen that the temperature of the soil at a distance from the frozen pipes is about 286.79 K (13.64°C) after 40 days of active freezing at a seepage velocity of 0, which is about 4.36°C lower than the original temperature and has less effect. The difference in soil temperature under different seepage velocity conditions was not significant for about the first 7 days and started to show significant differences after the 7th day, which was due to the fact that the observation point was farther away from the freezing tube, and with the increase of freezing time, the more distant soil started to show freezing walls at about the 7th day.

From Fig 12(C), it can be seen that the freezing effect of the soil at observation point 2 on path 4 in the upstream area decreases with increasing seepage velocity, and the soil forms a frozen curtain at about day 5.5 for a seepage velocity of 0. The soil can reach the temperature of 263.15 K (-10°C) required to form a tight and dense permafrost curtain for heads of water of 0, 0.2 m, and 0.4 m at the end of freezing. When the heads of water are 0.8 m and 1 m, the rebound phenomenon of the soil temperature at the end of freezing was due to the influence of seepage flow freezing pipes cold can not reach the area, so the temperature gradually increased. Fig 12(D) shows that the cooling effect is best when the heads of water is 0, but the end of freezing still does not reach the maximum temperature of 272.15 K (-1°C) for the formation of permafrost curtain, and the larger the heads of water is, the worse the cooling effect is.

**Fig 13:**
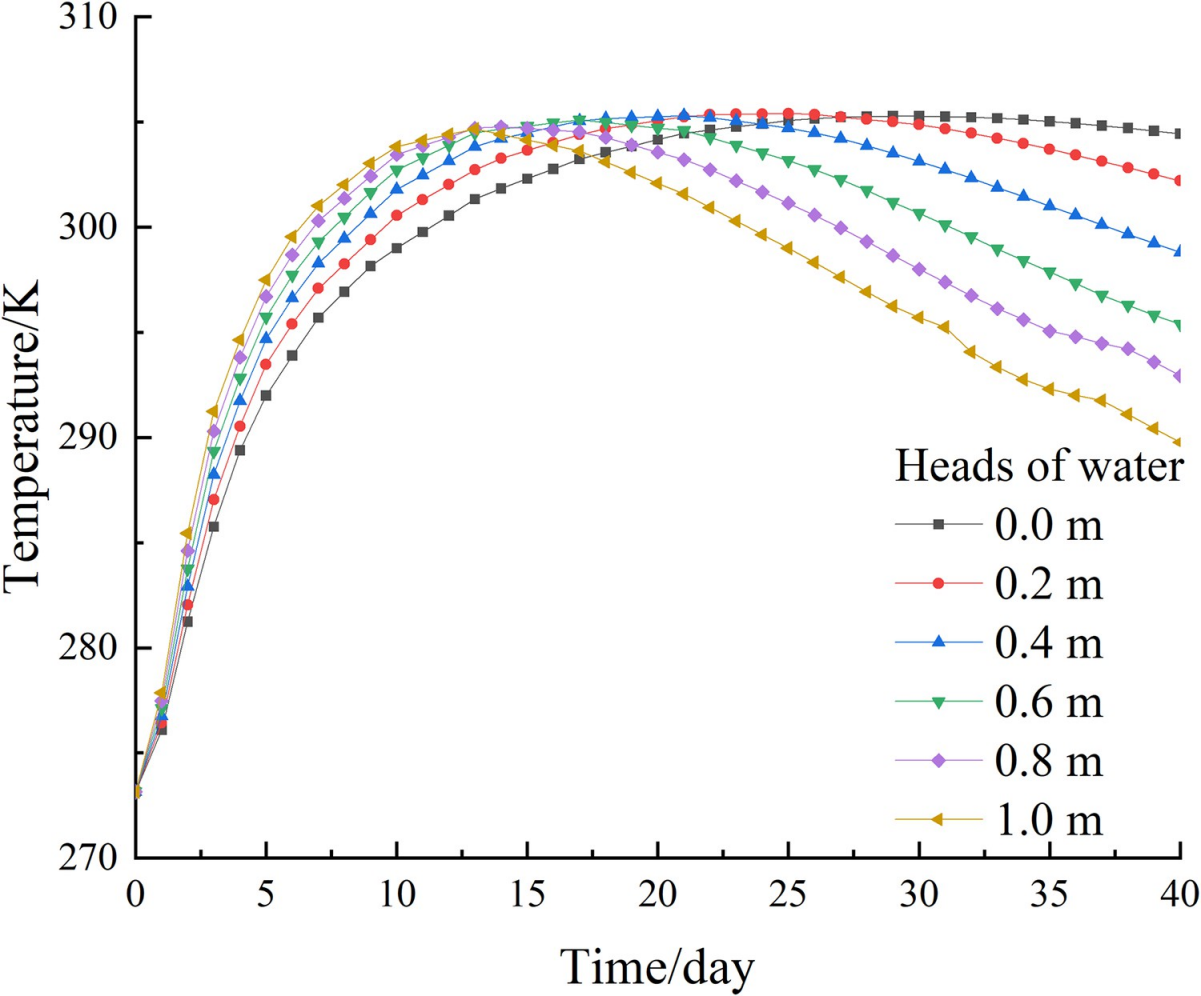
Temperature difference curve with different head s of water.

The temperature variation patterns of observation points 1 and 6 on path 2 under different heads of water conditions are shown in Fig 13. At the beginning of freezing, the difference between the temperatures of observation points 1 and 6 becomes larger and larger as the freezing time increases. When the freezing reaches a certain level, the difference between the two decreases gradually. Therefore, it can be concluded that in the process of freezing, there is a hysteresis in the influence of the seepage field on the temperature field, and the downstream area is affected later than the upstream area; the larger the heads of water, the larger the seepage velocity, and the more obvious the hysteresis.

**Fig 14:**
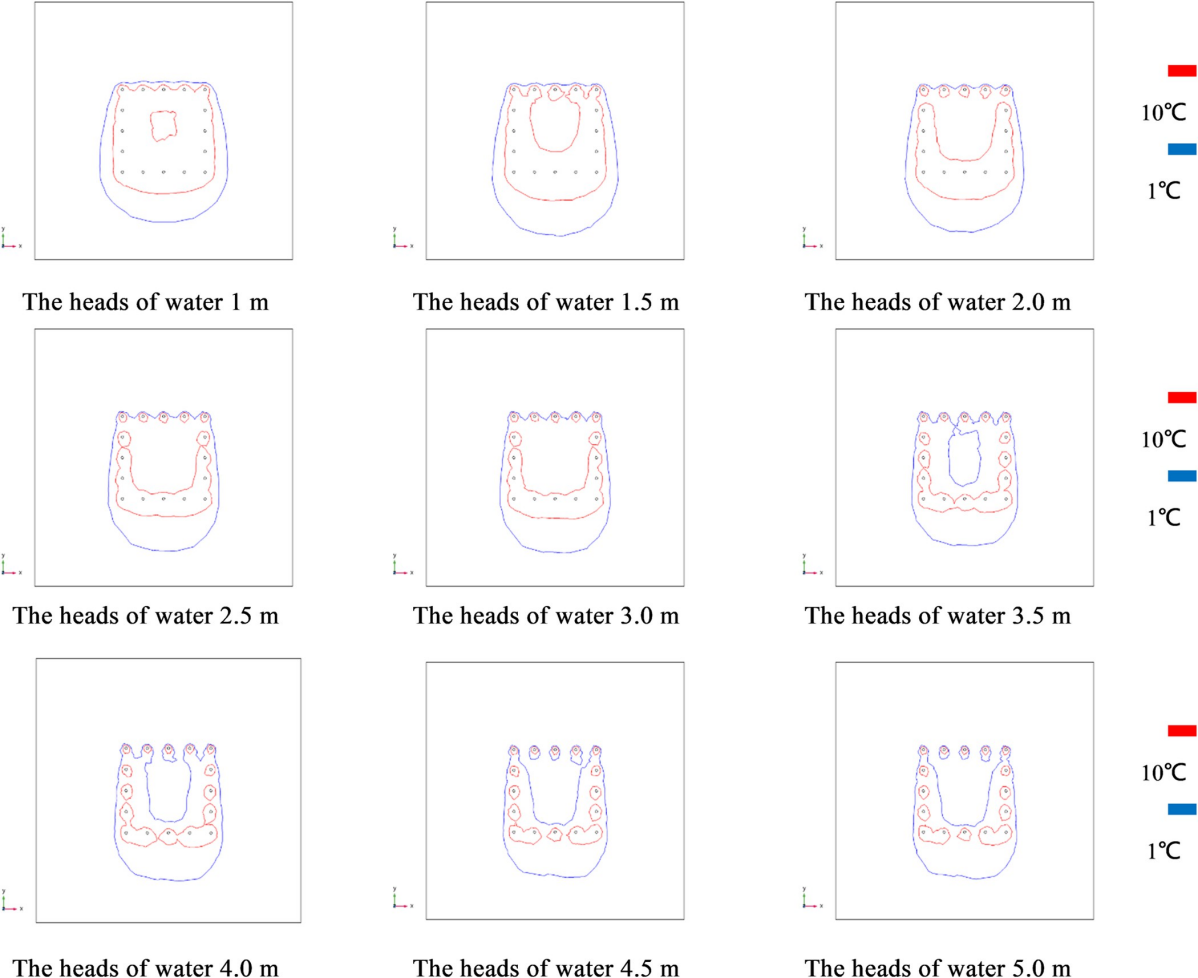
Isotherm at different heads of water for XY(Z = 3) cross section.

Since the 16 freezing pipes were arranged in a square pattern and the center of the device was not equipped with freezing pipes, the freezing efficiency of the soil at the center was the lowest. Observation point 9 on path 1 is at the lowest end of the freezing unit and has the worst freezing effect, so the XY (Z = 3) plane was selected for observation. The isotherm plot is shown in Fig 14. In Fig 10, it shows that when the heads of water is 3.5 m, the area with a temperature greater than 272.15 K (-1°C) appears for the first time in this plane, and the area cannot form a freezing curtain. With the increase of heads of water, the area where the freezing curtain cannot be formed gradually expands and the freezing effect becomes worse. Therefore, to ensure the freezing effect, the heads of water should be controlled from 1 m to 3.5 m, and the seepage velocity should be controlled from 1.65 m/d to 5.78 m/d.

## 6. Analysis of paths

From the above analysis, it can be seen that the larger the heads of water, the more obvious the influence of submarine groundwater on the temperature field. In order to further analyze the evolution of the temperature field of the new freezing device under a large flow rate, this section selects the case where the heads of water is 1 m for path analysis.

**Fig 15:**
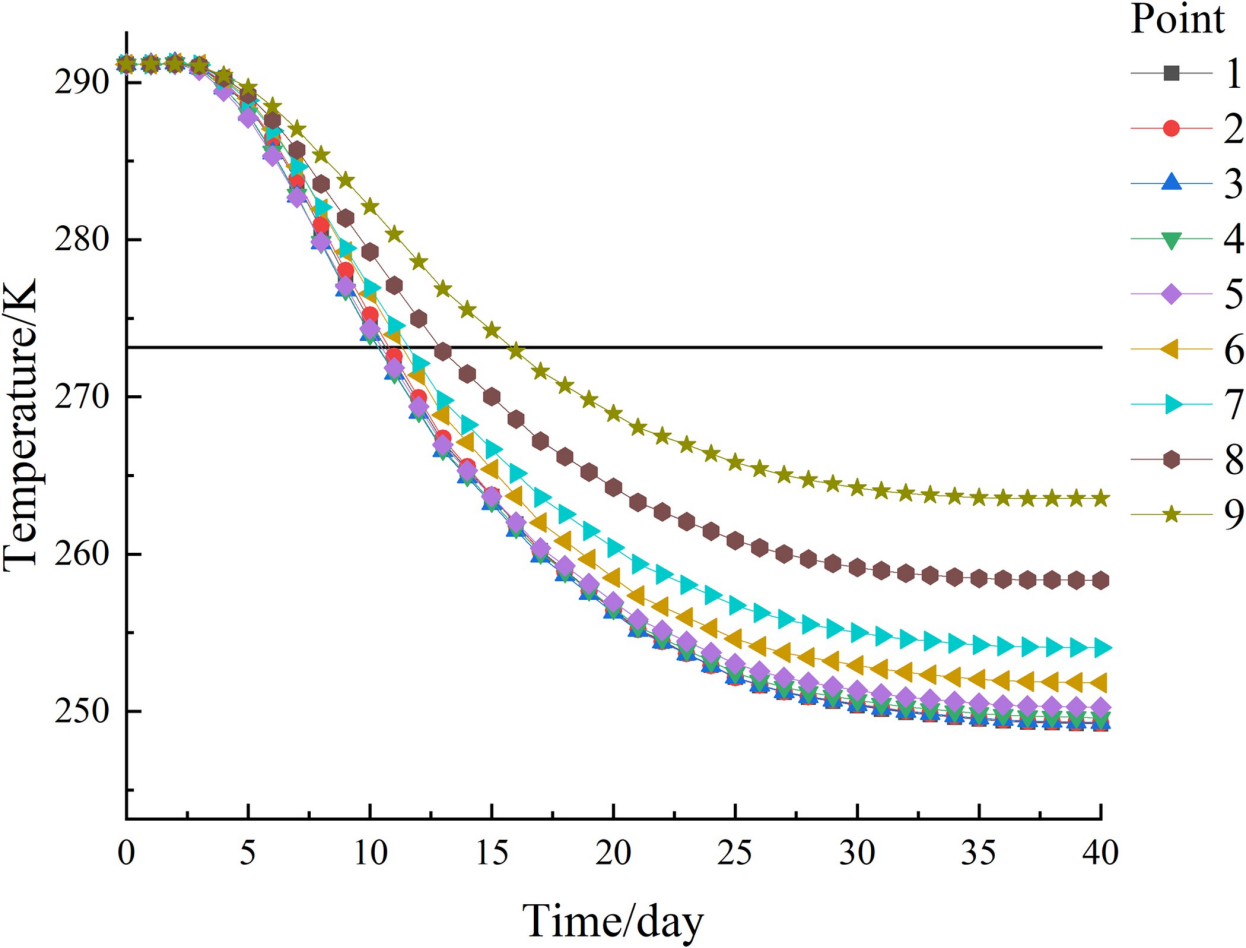
Path 1 temperature change curve of each observation point with time.

**Fig 16:**
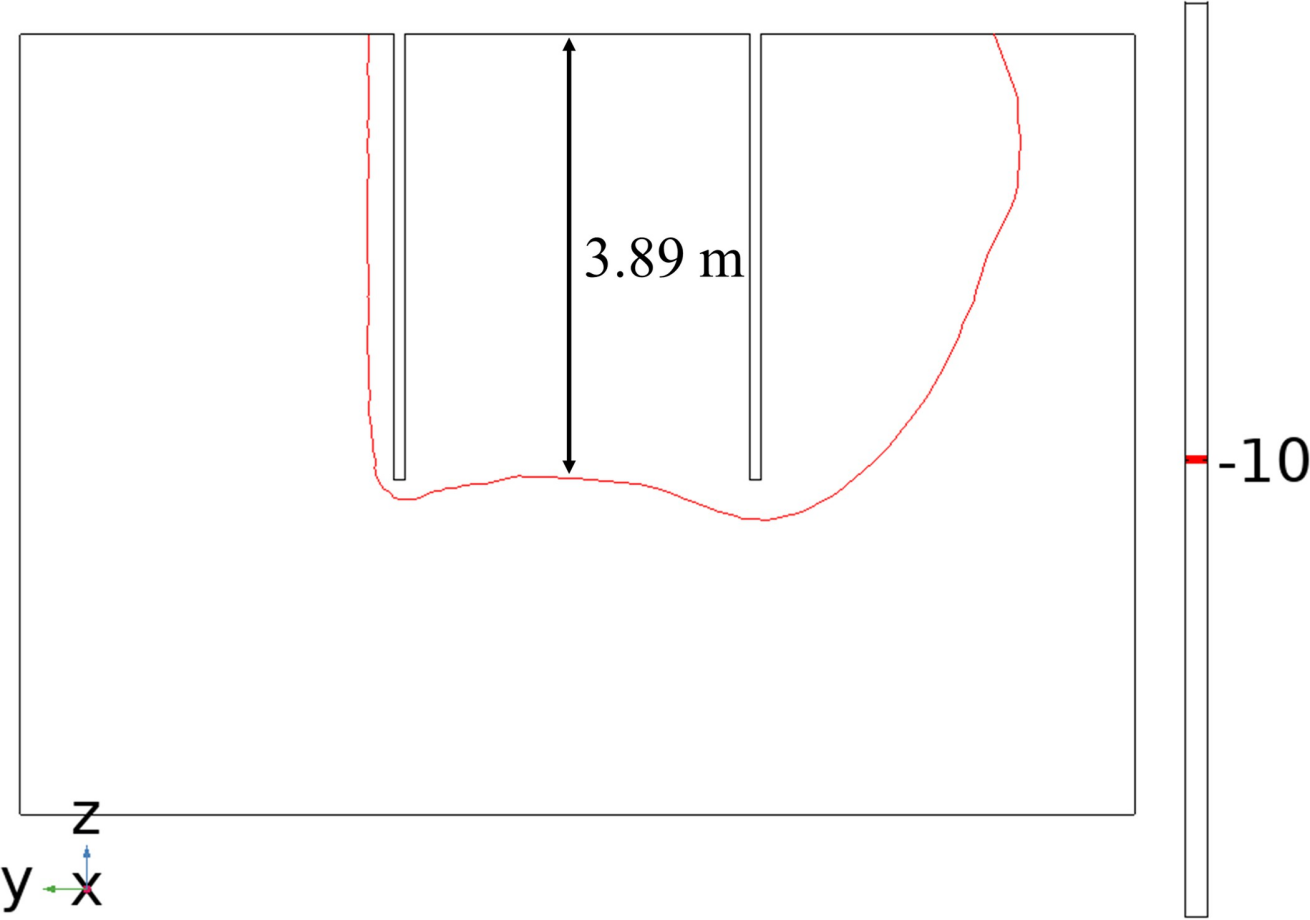
Thickness of permafrost curtain.

The freezing of each observation point on path 1 is shown in Fig 15. The temperature of each observation point at the end of freezing has a large difference, but the temperature is well below 272.15 K (-1°C), which can form a frozen curtain. In addition, the lowest temperature at the end of freezing was at observation point 1 (5, 5, 7) with 249.19 K (-23.96°C), forming a uniform and tight permafrost curtain. The highest temperature at the end of freezing was at observation point 9 (5, 5, 3) with a temperature of 263.53 K (-9.62°C), close to the temperature of 263.15 K (-10°C) required for the formation of a tight and dense permafrost curtain. The difference between the highest and lowest temperatures at the observation point is 14.34°C, and the central soil forms a stable and compact freezing wall with a thickness of about 3.89 m in the Z-axis direction, as shown in Fig 16.

**Fig 17:**
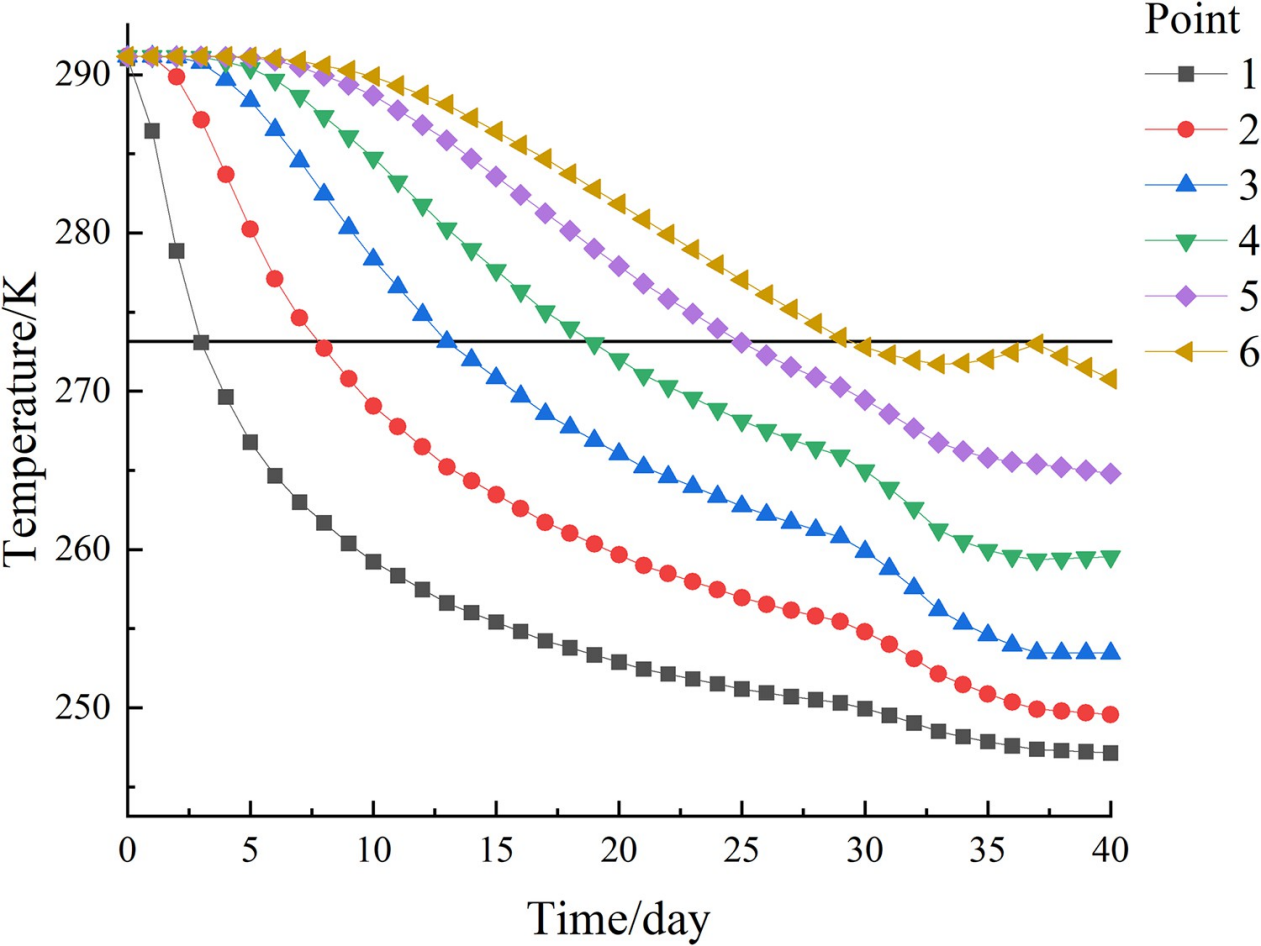
Path 2 temperature variation curve of each observation point with time.

The freezing situation of each observation point on path 2 is shown in Fig 17, and the temperature difference of each point on path 2 is more obvious after the end of freezing. The closer the area to the freezing pipes, the faster the cooling rate is, and the maximum cooling rate is about 6.685°C/d. In addition, the lowest temperature at the end of freezing is observation point 1, with a temperature of 247.15 K (-26°C), which can form a stable and compact permafrost curtain; the highest temperature at the end of freezing is observation point 6, with a temperature of 270.76 K (-2.39°C), which reaches the minimum temperature for the formation of permafrost curtain, and the difference between the highest and lowest temperature is 23.61°C.

**Fig 18:**
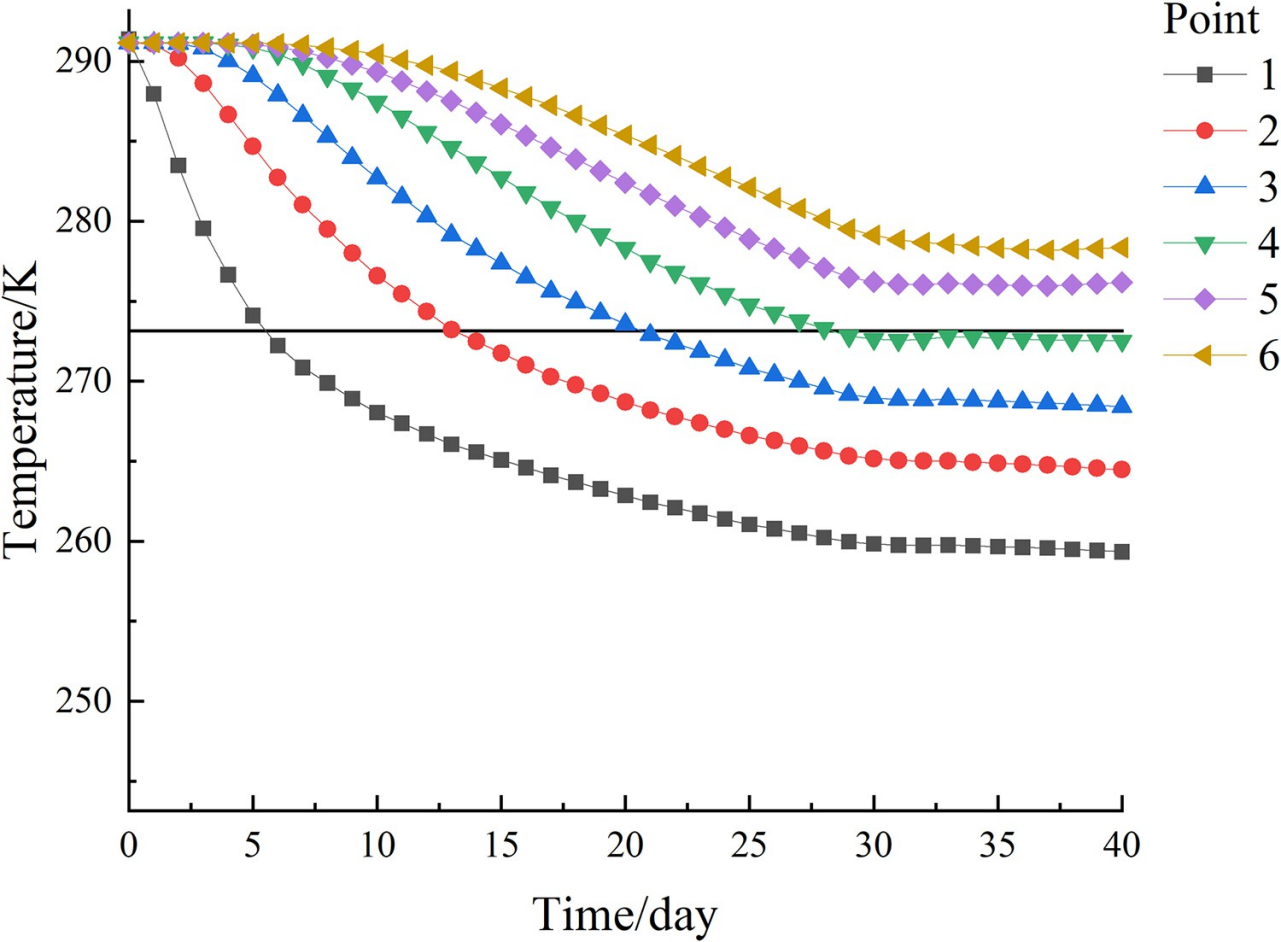
Path 3 temperature variation curve of each observation point with time.

The temperature change curve of each observation points on path 3 with time is shown in Fig 18. The temperature change of each point on path 3 at the end of freezing is similar to that of path 2. However, observation point 5 and observation point 6 did not reach a temperature below 273.15 K (0°C) at the end of freezing and no freezing occurred. Observation point 4 had a temperature of 272.52 K (-0.63°C) at the end of freezing, which did not reach the minimum temperature of 272.15 K (-1°C) for the formation of the frozen curtain. Observation point 1 reached the lowest temperature of 259.33 K (-13.82°C) at the end of freezing, allowing the formation of a tight and dense frozen curtain. The difference between the highest and lowest temperatures is 19.02°C.

**Fig 19:**
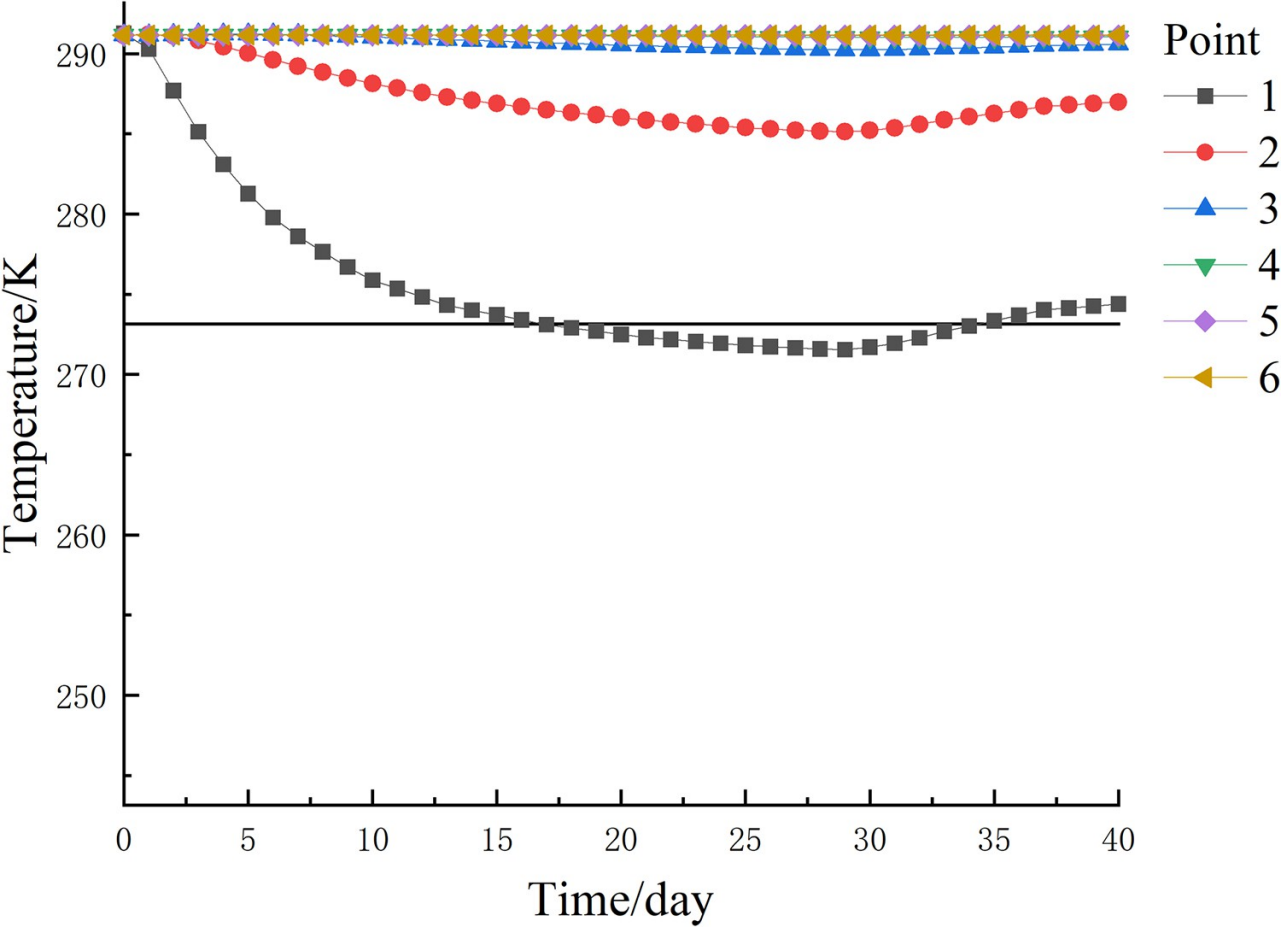
Path 4 temperature variation curve of each observation point with time.

**Fig 20:**
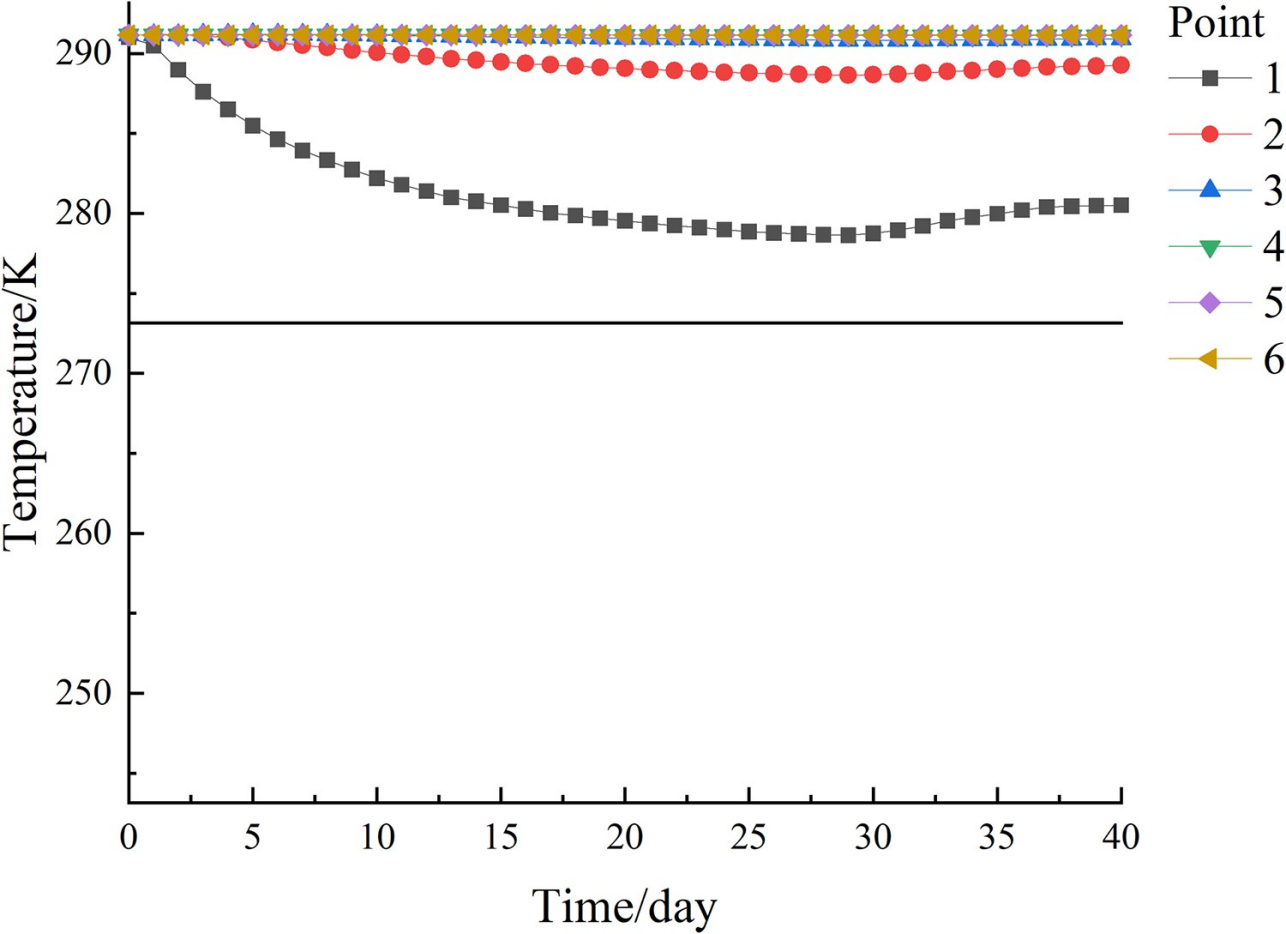
Path 5 temperature variation curve of each observation point with time.

The temperature variation curves with time for each observation point on path 4 are shown in Fig 19. The cooling effect is most obvious at observation point 1, where the lowest temperature of 271.55 K (-1.6°C) was reached on day 29 during the cooling process and a permafrost curtain was formed, but the temperature at this point was 274.39 K (1.24°C) at the end of freezing and the permafrost curtain melted. The observation point in this area is far from the freezing tube and the temperature change is not significant. The temperature variation curves with time at each observation point on path 5 are shown in Fig 20. The cooling effect was most pronounced at observation point 1, which reached a minimum temperature of 278.63 K (5.48°C) on day 29 during the cooling process, and the temperature at this point was 280.52 K (7.37°C) at the end of freezing. The temperature variation at the observed points in this region was similar to path 4. If the 263.15 K (-10°C) and 272.15 K (-1°C) isotherms are considered as freezing boundaries, and the points on the nearest isotherms on the X, Y and Z axes are taken as the maximum area for forming a frozen curtain, the freezing device can form a frozen wall of 3.70 m × 4.61 m × 4.12 m. The extent of forming a tight and dense frozen wall is 3.65 m × 4.20 m × 3.89 m.

## 7. Analysis and recommendations

The device is used in submarine reinforcement when the heads of water is greater than 1 m or the seepage velocity is greater than 1.65 m/d, the freezing effect of the soil in the center of the freezing device is good. A tight and dense frozen soil curtain comparable to the volume of the device can be formed after the end of freezing. When the heads of water is less than 1 m or the seepage velocity is less than 1.65 m/d the freezing effect of the central soil is not effective, but the frozen soil curtain thickness around the frozen pipe meets the requirements after the freezing is finished. When the heads of water exceeds 3.5 m or the seepage velocity is greater than 5.78 m/d, the temperature of the soil at the center of the end of the frozen pipes gradually rises, and eventually no freezing curtain can be formed.

It is necessary to know the local hydrogeological conditions before using this device for freezing construction. The freezing effect is best in the range of seepage velocity from 1.65 m/d to 5.78 m/d, and it is possible to carry out freezing and consolidation of sandy soil on the seabed. Moreover, the existence of the tank outside the device makes it less affected by ocean currents when reinforcement is carried out on the seabed, and it can quickly reach the required temperature for reinforcement, which is conducive to improving freezing efficiency.

## 8. Conclusion

In this paper, the hydrothermal coupling module in the finite element software COMSOL is used to investigate the influence law of the new freezing device on the temperature field in the process of submarine sand reinforcement under seepage conditions, and the evolution of the temperature field for different seepage velocities was explored by changing the heads of water, and the following conclusions were drawn:

During the freezing process, The greater the seepage rate, the more pronounced the asymmetry of the permafrost curtain.

During the freezing process, there is a hysteresis in the effect of the seepage field on the temperature field, with the downstream region being affected later than the upstream region. The larger the heads of water or seepage velocity, the more obvious the hysteresis is.

Due to the influence of seepage flow, the freezing effect was good in the middle area of the soil without the freezing pipes, and as the seepage flow rate increased, the freezing effect of the soil in the middle area became better.

To ensure the freezing effect, the groundwater seepage rate should be between 1.65m/d and 5.78m/d.

## Supporting information

S1 File (ZIP)
